# Unilateral biportal endoscopic foraminotomy and diskectomy combined with piezosurgery for treating cervical spondylotic radiculopathy with neuropathic radicular pain

**DOI:** 10.3389/fneur.2023.1100641

**Published:** 2023-04-11

**Authors:** Peng Zhang, Yanghui Jin, Bo Zhu, Mingfeng Zheng, Xiaozhang Ying, Qi Zheng

**Affiliations:** Affiliated Hangzhou Chest Hospital, Zhejiang University School of Medicine, Hangzhou, China

**Keywords:** cervical spondylotic radiculopathy, neuropathic radicular pain, unilateral biportal endoscopy, piezosurgery, foraminotomy and diskectomy

## Abstract

**Objective:**

Unilateral biportal endoscopy (UBE) represents a relatively recent development in minimally invasive spine surgery. This study aimed to evaluate the efficacy and safety of UBE foraminotomy and diskectomy combined with piezosurgery for treating cervical spondylotic radiculopathy (CSR) with neuropathic radicular pain.

**Methods:**

We retrospectively analyzed the outcomes in 12 patients with CSR who underwent UBE foraminotomy and diskectomy combined with piezosurgery. The intraoperative blood loss, operative time, visual analog scale (VAS) scores for the neck and arm, neck disability index (NDI) scores, and complications were recorded.

**Results:**

Postoperative VAS scores of the neck and arm and NDI scores were significantly improved. Additionally, a postoperative CT scan revealed adequate enlargement of the cervical canal and nerve root. No specific complications occurred during surgery and the immediate postoperative period.

**Conclusions:**

This primary study indicated that the UBE foraminotomy and diskectomy with piezosurgery is a promising technique for treating cervical spondylotic radiculopathy with neuropathic radicular pain.

## Introduction

With the arrival of an aging society, the number of patients with cervical spondylotic radiculopathy (CSR), which is one of the degenerative spinal diseases, is increasing rapidly (1). CSR is the most common form of cervical spondylosis, mainly due to cervical nerve root compression caused by disc degeneration, herniation, segmental instability, or other conditions, mainly manifested as neuropathic radicular pain. CSR, the most prevalent form of cervical spondylosis, is caused by cervical nerve root compression due to disc degeneration, herniation, segmental instability, or other conditions, mainly manifested as neuropathic radicular pain (2). Although anterior cervical discectomy and fusion (ACDF) is the gold standard surgical strategy for the treatment of CSR, it has several disadvantages, including adjacent segment degeneration, incision and implant-related complications, and high hospitalization costs (3). In addition, it is reported that anterior open surgery is closely associated with a high risk of postoperative dysphagia (4). Consequently, minimally invasive spinal surgeries have emerged with similar clinical outcomes, which have several advantages, including less injury, shorter hospital stay, and fewer postoperative complications (5).

Unilateral biportal endoscopy (UBE) is a minimally invasive spinal surgery with minimal injury to the surrounding structures, thereby greatly increasing the stability of the operated segment (6). Currently, UBE has gradually been applied for cervical disorders with remarkable efficiency and conveniency (6, 7).

However, whether piezosurgery can be used in UBE surgery has not been reported. This study aimed to evaluate the efficacy and safety of UBE foraminotomy and diskectomy combined with piezosurgery for treating CSR with neuropathic radicular pain

## Materials and methods

### Clinical data

We retrospectively followed a group of 12 patients suffering from CSR and received UBE foraminotomy and diskectomy with piezosurgery between May 2020 and June 2021. The average age of patients was 50.2 ± 9.5 years, seven of them were men and five were women. The demographic and clinical characteristics of patients were shown in Table 1. This research was approved by the Ethics Committee of Affiliated Hangzhou Chest Hospital, Zhejiang University School of Medicine. Each patient provided written informed consent before being administered any treatment.

**Table 1:** Biodemographic data of patients with CSR who underwent UBE foraminotomy and diskectomy combined with piezosurgery.

**Variables**	**Mean (SD)**	**Frequency (%)**
**Age (years)**		
30–39		1 (8.3)
40–49		4 (33.3)
50–59		5 (41.7)
60–70		2 (16.7)
**Gender**		
Male		7 (58.3)
Female		5 (41.7)
**Chief complaint**		
Neck pain		2 (16.7)
Arm pain		4 (33.3)
Both		6 (50.0)
**Level, No**.		
C4–C5		3 (25.0)
C5–C6		4 (33.3)
C6–C7		5 (41.7)
Operative time (min)	251.2 (14.6)	
Blood loss (ml)	50.2 (9.5)	
Follow-up (month)	16.8 (1.8)	

The CSR patients were initially diagnosed according to a combination of the patient's clinical history, radicular symptoms, and radiographic scan. Inclusion criteria were as follow: no improvement in radicular symptoms after at least 3 months of conservative treatments with oral NSAIDs and neurotrophy drugs, no previous cervical surgery, 1-level disk herniation, no large disk repress cervical myelopathy, and the patient refused conventional open surgeries. Exclusion criteria included: disc herniation with no radicular symptoms, accompanying spinal instability, and coexisting spinal infection or malignancy.

### Surgical procedure

Following general anesthesia, the patient underwent surgery in a prone position on a chest pad, and the neck was placed in slight flexion using a horseshoe-shaped headrest. With the guidance of a C-arm fluoroscope, the target interlaminar space was identified and marked. Two 1.5 cm skin incisions about 1 to 1.5 cm ipsilateral to the spinous process line were created at 1 cm above and below the marker line. Next, a series of dilators were used to split the paraspinal muscle and created the two viewing and working portals. The viewing portal was used to provide surgical vision, while the working portal was used for instrument handling.

**Figure 1:**
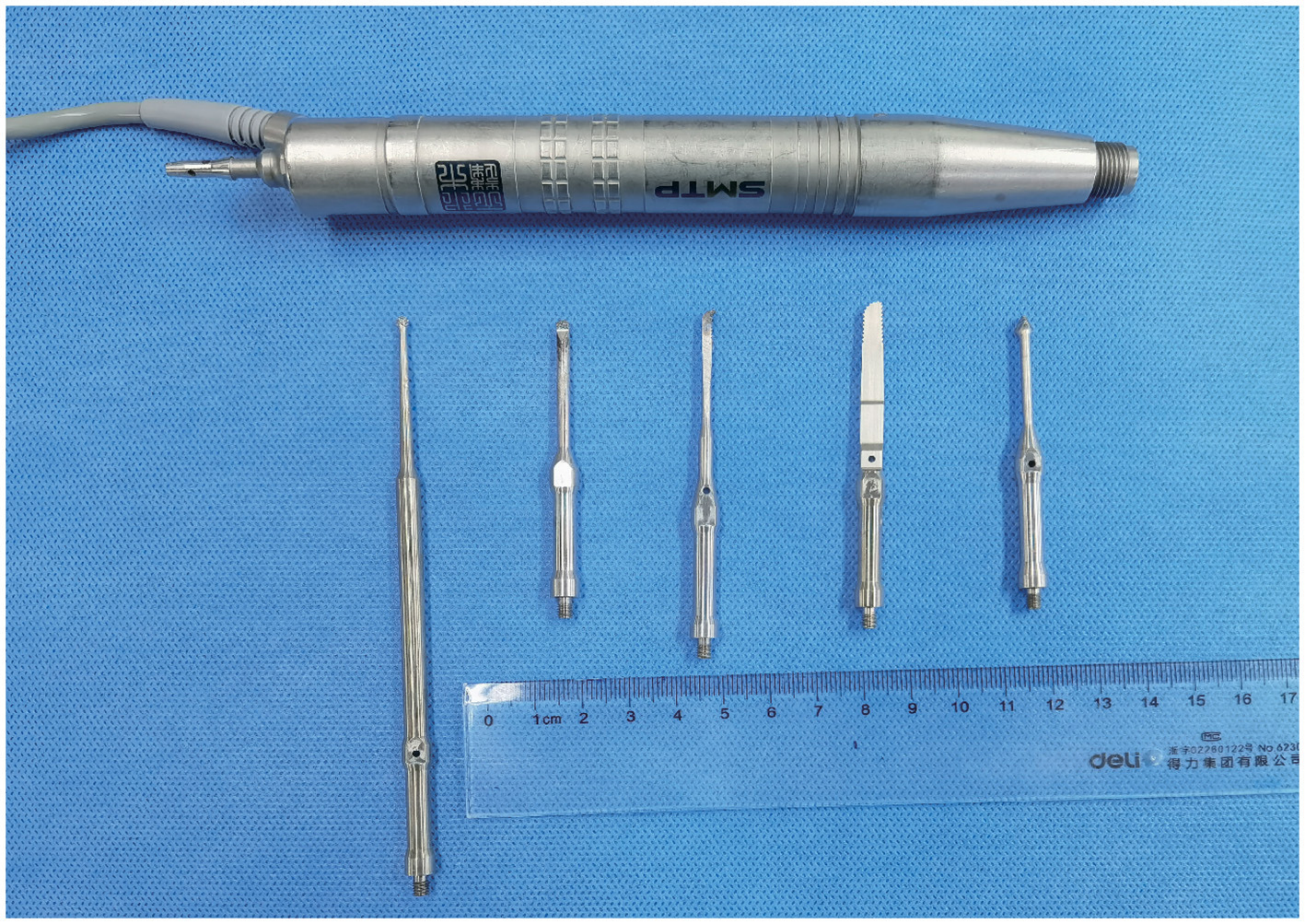
SMTP Piezosurgery device with hand-piece and different inserts.

Under endoscopic guidance, the soft tissue was removed from the bone surface with a bipolar radiofrequency probe until the “V” point is exposed. Thereafter, foraminotomy was carefully performed on the inferior lamina, superior lamina, interlaminar spaces, and the medial portion of the facet joint. An oval hole about 2 cm in the bone was made with a high-speed spherical drill and Piezosurgery device (XD880A, SMTP Technology Co., Ltd.) (Figure 1). The use of Piezosurgery device can safely and efficiently remove the bone around the nerve root, thereby fully releasing the nerve root.

**Figure 2:**
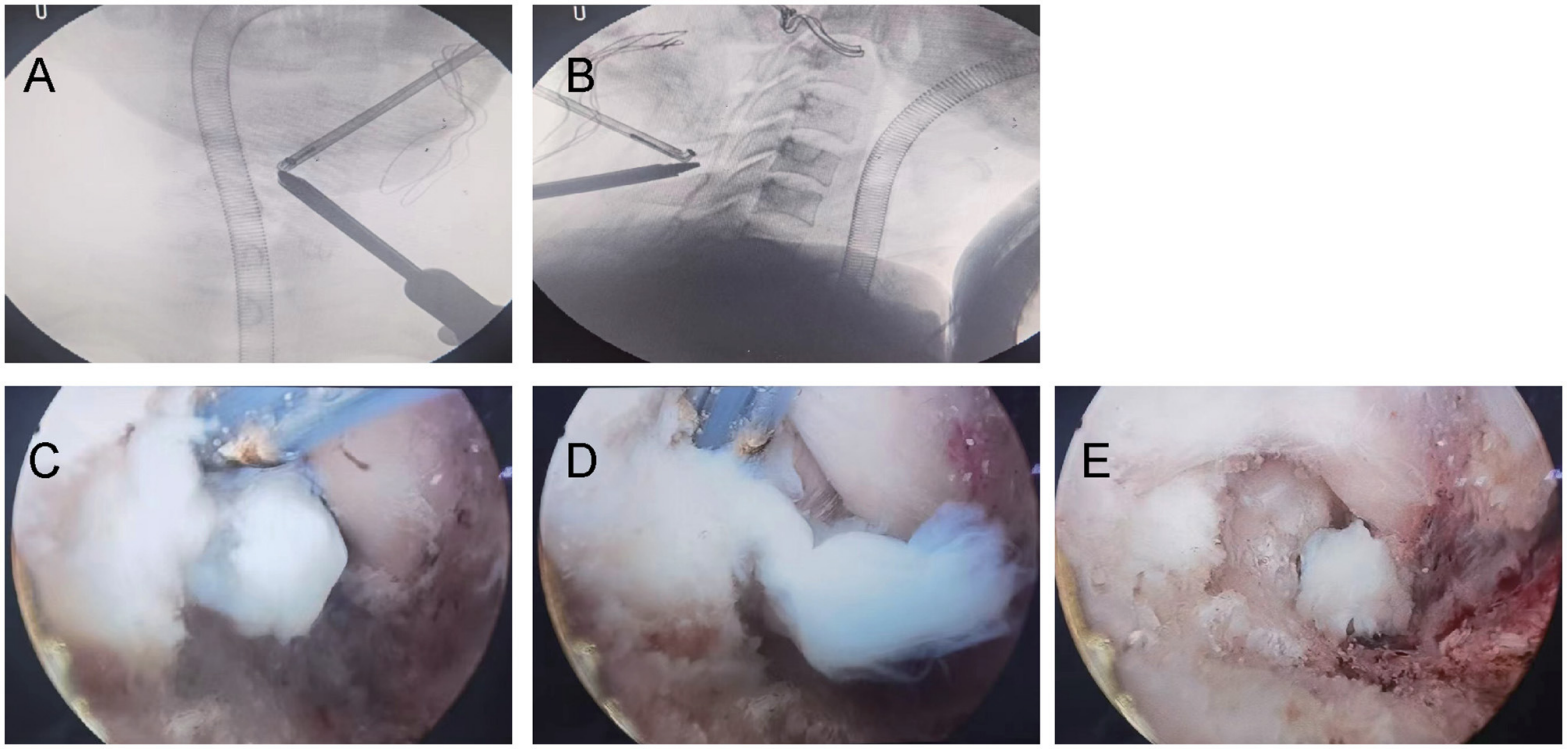
**(A, B)** Radiographic anteroposterior represented the locations of viewing and working portals. **(C–E)** Diskectomy was performed.

After the cervical nerve root and herniated disc were seen under the endoscope, a diskectomy was performed to remove the herniated nucleus pulposus tissue, thus releasing the nerve root. Subsequently, complete decompression of the spinal cord and nerve root was confirmed. Finally, the endoscopy and instrument were withdrawn, a drainage tube was placed, and then the wound was closed. The surgical procedure is shown in Figure 2.

### Evaluation

Intraoperative blood loss, operative time, visual analog scale (VAS) score for the neck and arm, Neck Disability Index (NDI) scores and complications were documented. Radiographic examinations including MRI and CT were performed to access the extent of disc herniation, the status of the spinal cord and nerve root, and relevant bony structures preoperatively and at the follow-up periods.

### Statistical analysis

Statistical analyses were conducted on all data employing SPSS 16.0 (IBM, USA). Results were presented as mean ± standard deviation (SD). Herein, Fisher's exact probability test, independent-samples *t*-test, and one-way ANOVA analysis were conducted employing SPSS. *p* < 0.05 was judged significant.

## Results

**Figure 3:**
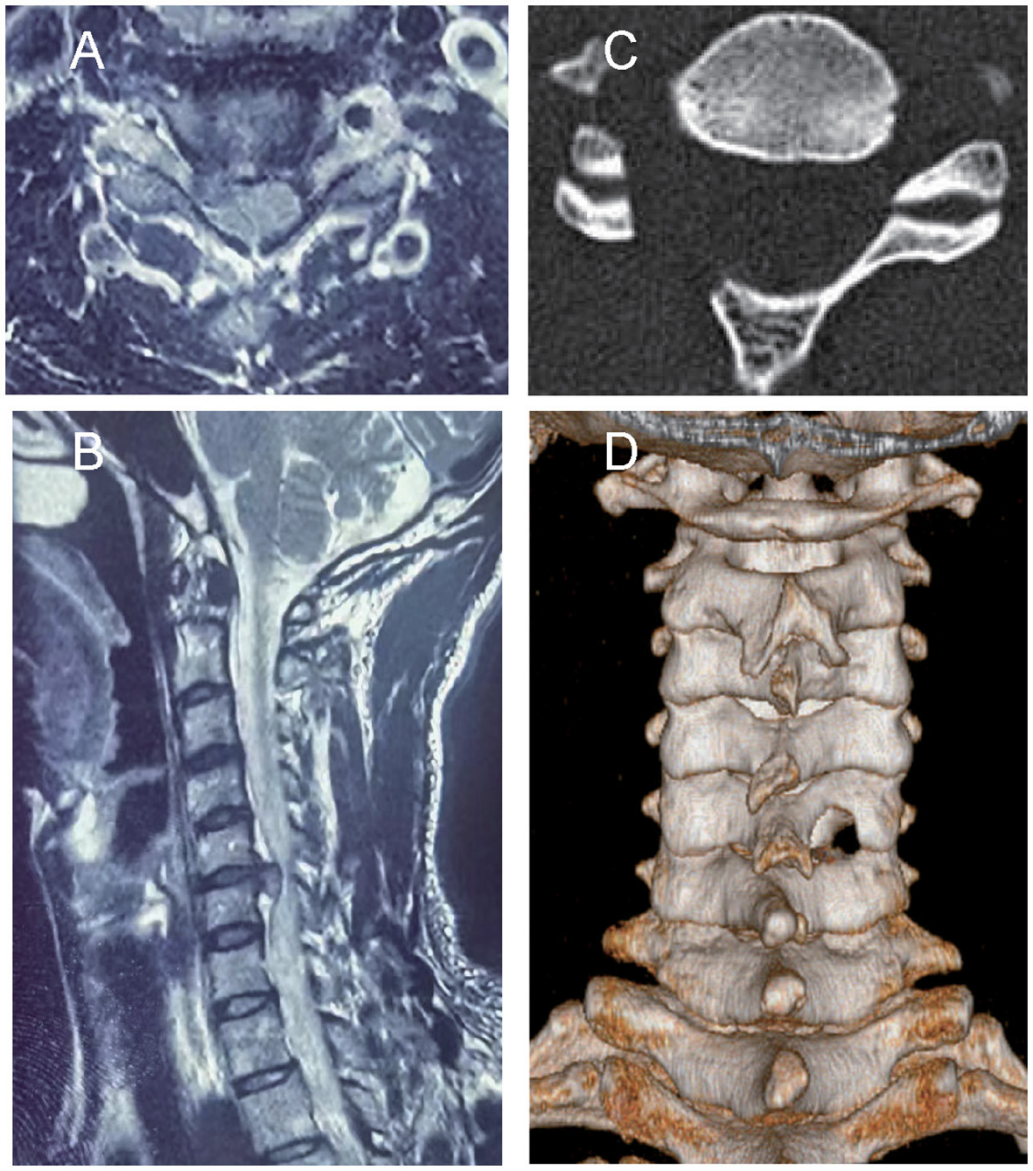
A 40-year-old man had cervical 5–6 intervertebral disc herniation, presenting with neck pain and numbness in the right upper limb. **(A, B)** Preoperative axial and sagittal magnetic resonance images showed C5/6 disk protrusion on the right subarticular zone. **(C)** Postoperative axial computed tomography scan showed the location of the unilateral foraminotomy. **(D)** Postoperative 3-dimensional computed tomography scan showed widening of the spinal canal after surgery.

**Figure 4:**
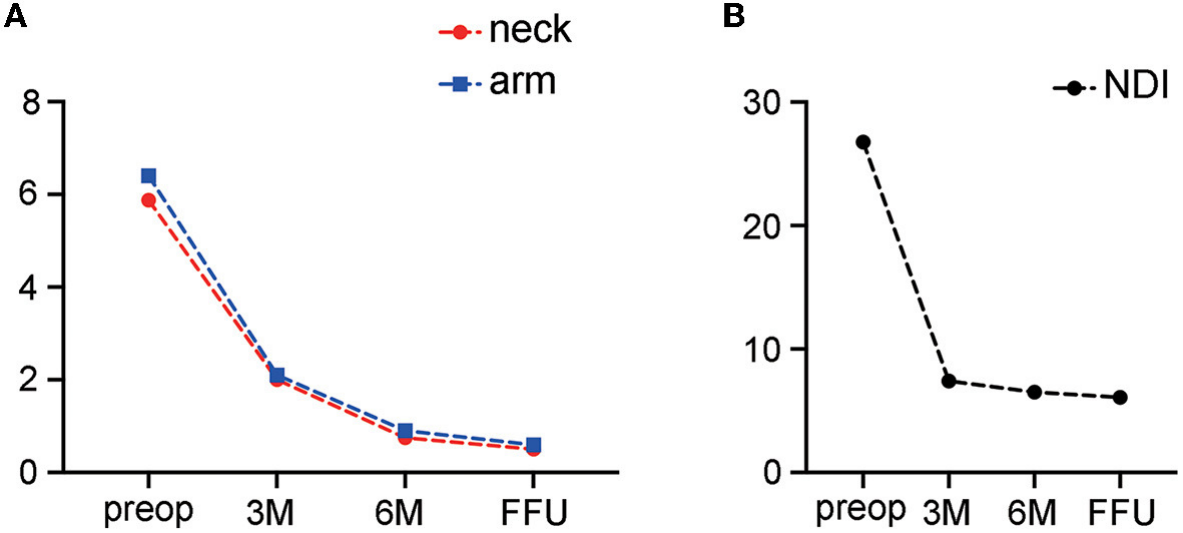
At the final follow-up, VAS scores of the neck and arm **(A)** and the Neck Disability Index (NDI) scores **(B)** were significantly improved compared to the pre-operation (*P* < 0.05). preop, preoperative; M, month; FFU, final follow-up.

Table 1 shows the mean operation time and intraoperative blood loss. Postoperative CT scan revealed adequate enlargement of the cervical canal and nerve root (Figure 3). At the final follow-up, VAS scores of the neck and arm and NDI scores were significantly improved compared to the pre-operation (Figure 4).

No specific complications occurred during surgery and the immediate postoperative period. No patient required additional surgery for sustained or aggravated symptoms during postoperative hospitalization. No patient had the nerve or vascular injury during surgery, and no patient experienced persistent or aggravated symptoms during the postoperative hospitalization.

## Discussion

CSR, mainly manifested as neuropathic radicular pain, is one of the common spinal disorders and usually requires only conservative treatment. However, when conservative treatment fails, surgery is necessary to relieve the symptoms by removing the compression (8). Generally, ACDF is the primary surgical strategy for CSR with good outcomes, however, it is accompanied by several limitations, including limited neck mobility, adjacent segment degeneration, and high reoperation rate (9–11). In addition, there is conflicting evidence regarding the efficacy of anterior foraminotomy, with reported success rates of 52–99%, but recurrence of radicular symptoms is as high as 30% (12).

With the development of new technology and devices, the application of minimally invasive endoscopic surgery to treat CSR is gradually increasing, with better outcomes and fewer complications compared to ACDF (13–15). A decade ago, percutaneous endoscopic cervical diskectomy using a small-sized single portal was introduced, which was reported to not only minimize surgical trauma but also effectively relieve compression (16, 17). However, working and viewing in the same narrow portal manifested several difficulties, such as disorientation and difficulty in maneuvering (18, 19).

Currently, UBE technology represents a relatively recent development in minimally invasive spine surgery (20, 21). Compared with uniportal endoscopic surgery, UBE with two portals has important advantages in improving the scope of the surgical field and the freedom of working devices. Through independent viewing and working portals, the operator can maneuver comfortably with working devices, such as piezosurgery and drills, to expand the range of exploration and decompression. Moreover, independent control of the 30-degree endoscope can enlarge the surgical field of view, which is conducive to the observation of tiny blood vessels and ligaments, thereby reducing iatrogenic blood vessels and neurological injuries. At the same time, the use of continuous irrigation not only provides a clearer view to preventing intraoperative complications but also flushes away inflammatory factors to reduce postoperative pain and the risk of infection (22).

As we all know, the vessels and nerves around the spine are dense and complex. During foraminotomy and diskectomy, there is a risk of unintended injury to the nerve root, blood vessel, and dura. In particular, rotary drills are commonly used to remove bone tissue, however, they undoubtedly increase the risk of neurovascular structures due to their rotational power and limited operating space (23). Piezosurgery technique is based on micro-vibrations, which selectively cuts bone tissue and preserve surrounding soft tissue structures (24). Piezosurgery device performs bone cutting by controlled scraping motion without rotational power, which provides precise and safe bone removal procedures with reduced risk to adjacent soft tissue (25). To our knowledge, the present study first combined Piezosurgery device and UBE for cervical foraminotomy and discectomy to optimize surgical precision and reduce surgical risks. A series of foraminotomy and diskectomy in 12 patients with CSR was performed, which showed that the postoperative VSA and NDI scores were significantly improved at the final follow-up. No nerve or vascular injury occurred during surgery and the immediate postoperative period. Meanwhile, no patient experienced persistent or aggravated symptoms during the postoperative hospitalization.

### Limitations

The current study has several limitations: (1) It was a retrospective non-randomized comparative study; (2) Need a larger number of patients and a longer follow-up period to further confirm the outcomes; (3) The disadvantage of UBE surgery is that it is relatively difficult and requires a long learning curve for the surgeon to gain proficiency.

### Conclusions

In summary, UBE foraminotomy and diskectomy with piezosurgery is a promising technique for the treatment of cervical spondylotic radiculopathy, which has several advantages, including improvement of pain and functional disability, less blood loss, low hospitalization cost, and preservation of spinal tissue.

## Data availability statement

The raw data supporting the conclusions of this article will be made available by the authors, without undue reservation.

## Ethics statement

The studies involving human participants were reviewed and approved by the Ethics Committee of Affiliated Hangzhou Chest Hospital, Zhejiang University School of Medicine. The patients/participants provided their written informed consent to participate in this study.

## Author contributions

Conceptualization: YJ. Methodology: BZ. Validation: MZ. Writing original draft preparation: PZ. Writing-review and editing: XY. Project administration: QZ. All authors contributed to the article and approved the submitted version.
